# Perspectives on the teaching of Occupational Dentistry according to university curricula in Southeast Brazil

**DOI:** 10.47626/1679-4435-2020-707

**Published:** 2023-02-03

**Authors:** Danielle Fernandes Lopes, Luan Viana Faria, Yuri de Lima Medeiros, Daniella Guedes de Figueiredo Lopes, Celso Neiva Campos

**Affiliations:** 1 Departamento de Clínica Odontológica, Universidade Federal de Juiz de Fora, Juiz de Fora, MG, Brazil; 2 Programa de Pós-Graduação em Odontologia, Faculdade de Odontologia de Araraquara, Universidade Estadual Paulista, SP, Brazil

**Keywords:** occupational dentistry, curriculum, dentistry education, odontologia do trabalho, currículo, educação em odontologia

## Abstract

**Introduction:**

Occupational Dentistry is a specialty recently acknowledged by the Federal Council of Dentistry that seeks to prevent work-related oral health issues. It aims to improve workers’ quality of life and promote a more efficient productive development.

**Objectives:**

This study aimed to investigate whether the subject of Occupational Dentistry was included in the curriculum of undergraduate Dentistry courses in Southeast Brazil.

**Methods:**

The curriculum of universities registered on the Brazilian Ministry of Health’s website (e-MEC) were analyzed regarding type of university administration (private or public), inclusion of Occupational Dentistry in the curriculum of Dentistry courses, whether the subject was compulsory or not, and subject workload. Universities that did not make the course curriculum available on their website were excluded from the analysis.

**Results:**

Of 176 universities registered on e-MEC, 144 were included in the study. Most universities (86.9%) were private, whereas only 13.1% were public. Occupational Dentistry was available in 10 universities. The subject was compulsory in 4 and optional in another 4 universities, with a total mean workload of 37.5 hours. Two universities did not disclose this information.

**Conclusions:**

Our analysis allowed the investigation of the overall inclusion of Occupational Dentistry in the curriculum of Dentistry courses in Southeast Brazil. Only a small percentage of universities (6.9%), mostly private, included the subject in the course curriculum, usually on a compulsory basis.

## Abstract (pt)

**Introdução:**

A Odontologia do Trabalho é uma especialidade recentemente reconhecida pelo Conselho Federal de Odontologia e ocupa-se em combater agravos à saúde bucal do trabalhador decorrentes de seu ambiente de trabalho. Ela busca melhorar a qualidade de vida do profissional e propiciar o desenvolvimento produtivo mais eficiente.

**Objetivos:**

Este trabalho se propõe a apresentar um panorama da disciplina de Odontologia do Trabalho nas instituições de ensino superior que possuem o curso de graduação em Odontologia na região Sudeste do Brasil.

**Métodos:**

Foi realizada uma análise das grades curriculares de todas as instituições cadastradas no portal eletrônico do Ministério da Educação (e-MEC), analisando quantas eram privadas e públicas, quais tinham a disciplina, se era obrigatória ou optativa e a carga horária. Foram excluídas da análise as instituições que não disponibilizaram a grade curricular em seus sítios eletrônicos.

**Resultados:**

Foram encontradas 176 instituições, sendo que, destas, 144 foram incluídas no estudo. Entre as incluídas, 86,9% foram particulares e 13,1% foram públicas. A disciplina foi encontrada em 10 instituições, sendo que é ofertada como obrigatória em quatro e como optativa em outras quatro, totalizando uma carga horária média de 37,5 horas, desconsiderando duas universidades que não forneceram essas informações adicionais.

**Conclusões:**

A partir desta análise, foi possível traçar um cenário da inserção da Odontologia do Trabalho, demonstrando que a oferta da disciplina se encontra bastante reduzida (6,9%), sendo mais prevalente em universidades privadas e de forma obrigatória.

## INTRODUCTION

Occupational Dentistry (OD) is a specialty that seeks to prevent work-related oral diseases and was recently acknowledged by the Brazilian Federal Council of Dentistry (Conselho Federal de Odontologia, CFO).^1^ OD aims to improve workers’ quality of life and, consequently, promote a more efficient productive development, minimizing the risks of complications and accidents.^2^

The specialty was regulated by CFO Resolution no. 22, effective as of December 27, 2001, article 4. Its attributions are described in section X of article 30: “the goal of Occupational Dentistry is to find a way to make the preservation of oral health compatible with occupational activities.”^3^ According to Resolution no. 25/2022, OD comprises the following areas of competence: “I) identification, assessment, and surveillance of environmental factors that may pose a risk to oral health in the workplace, at any stage of the production process; II) technical advice and health care; III) planning and implementation of campaigns and programs to educate workers; IV) statistical organization of oral-related morbidity and mortality; and V) investigation of possible associations between occupational activities and oral diseases and conduction of dental examinations for labor purposes.”^4^

Oral diseases are known to have possible negative effects on people’s well-being, which may compromise their work performance and cause behavioral disturbances. Occupational, mechanical, chemical, and physical exposures are thus associated with oral changes such as dental caries, tooth pain, dental erosion, periodontal disease, mucosal injury, and salivary alterations, which can drastically affect workers’ oral health.^5^

Therefore, the inclusion of OD in the curriculum of undergraduate Dentistry courses has been increasingly discussed, given that industrial and technological advances predispose workers to changes in their oral health, and dentists should be able to promote prevention and monitoring of oral health issues in the work environment.^6^

The objective of this cross-sectional study was to investigate whether undergraduate Dentistry courses in the Southeast region of Brazil have included OD in their curriculum.

## METHODS

Study methodology is described in detail in Chart 1. Because this was a cross-sectional, documentary study with publicly available data, approval from the Research Ethics Committee was not required.

**Chart 1:** Study methodology

Focus of the study	The search for undergraduate Dentistry courses registered on the Brazilian Ministry of Education’s website (e-MEC) located in Southeast Brazil.
Selection criteria	Inclusion criteria: universities with undergraduate Dentistry courses registered on e-MEC with some type of electronic communication (the website being the main one). Exclusion criteria: universities that did not make the course curriculum available on their website.
Study variables	Type of university administration (public or private); inclusion of OD as a subject in the curriculum of Dentistry courses; whether the subject was compulsory or not; allocated hours.
Data analysis and tabulation	Data tabulation was conducted using GraphPad Prism 8.1.2 (GraphPad Software Inc., La Jolla, USA); statistical analysis and interrelation of data were conducted by analyzing study variables according to included states and type of university administration.

The universities included in this study were selected using the Brazilian Ministry of Education’s website (e-MEC), which contains a list of universities regularly registered with the Brazilian Ministry of Education.^7^ The Southeast region of Brazil was selected as the target study population because it has a higher demographic density compared with other regions of the country. The list of universities was obtained from the e-MEC system on March 6, 2019.

The search was conducted from March to June 2019. When the course curriculum was not available in the university’s website, an email was sent to the course coordinator. A maximum period of 2 months was stipulated for receiving a response. If a response was not received, the university was automatically excluded from the study.

## RESULTS

**Chart 3:**
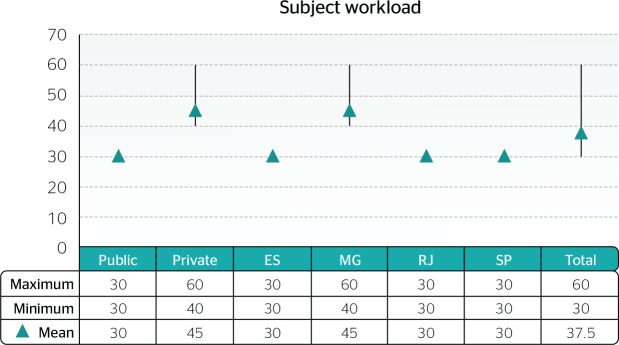
Occupational Dentistry workload according to type of university administration and state

Overall results are shown in Chart 2. Most (60%) of the universities that included OD in the curriculum of Dentistry courses were private. The state of Minas Gerais had the highest number of universities with the subject, accounting for 40% of all universities. Table 1 shows the detailed distribution. The subject was compulsory in 4 universities, optional in another 4 universities, and 2 universities did not disclose this information. Eight universities disclosed the allocated hours for the subject, with a total mean of 37.5 hours (SD = 10.35). Private universities and the state of Minas Gerais allocated the highest number of hours (Chart 3).

**Chart 2:** Results after applying the eligibility criteria

Focus of the study	176 universities were regularly registered on the Brazilian Ministry of Education’s website.
Eligibility criteria	144 universities were selected for the study, of which 32 were excluded because the course curriculum was not available on their website or because the course coordinator did not respond to our email within the specified time.
Data analysis and tabulation	The subject was available in 10 universities, and university variables were tabulated and analyzed using absolute and relative frequencies.

ES = Espírito Santo; MG = Minas Gerais; RJ = Rio de Janeiro; SP = São Paulo.

**Table 1:** Distribution of universities in Southeast Brazil by state, type of administration, and subject characteristics

University	Type of administration	Compulsory or optional	Type of approach
Espírito Santo			
1	Public	Compulsory	Theoretical
2	Private	-	-
Minas Gerais			
3	Private	Compulsory	Theoretical
4	Private	Compulsory	Theoretical
5	Private	Compulsory	Theoretical
6	Private	Optional	Theoretical
Rio de Janeiro			
7	Public	Optional	Theoretical
São Paulo			
8	Private	-	-
9	Public	Optional	Theoretical
10	Public	Optional	Theoretical

Subject nomenclature varied according to different universities, including: “Workers’ Health and Legislation”, “Occupational Dentistry”, “Topics in Occupational Dentistry”, and “Occupational and Legal Dentistry” (combined with Legal Dentistry).

## DISCUSSION

Work-related health issues are assessed by medical history and physical examination, as well as visits to the workplace. Assistance from the aggravated worker and collaboration from the employee, colleagues, and the government are essential for investigation and preventive intervention. A key feature is the need to investigate exposure with a multidisciplinary investigation team that can assess which workplace characteristics are necessary for quantitative assessment of the degree of exposure, interpretation of results, and development of preventive guidelines.^8^

OD is a relatively new specialty that seeks to prevent oral health issues in workers. It can be performed in several work environments, aiming to identify potential risks and educate workers as a way to promote health in the work environment, involving social, economic, cultural, educational, and behavioral aspects.^1^

Health professionals are prone to occupational diseases because they are continuously exposed to infectious waste and sharps, among other unhealthy factors in the workplace.^9^ Occupational accidents occupy a prominent position in this context, as they can cause health problems and interfere with the health-disease process.^10^

According to Silva Junior et al.,^11^ implementing policies focused on the prevention of infectious diseases, especially occupational diseases, for health students is urgently needed, since these students are directly exposed to biological material.

Despite the fact that OD became a specialty in 2001, many academics are unaware of its existence, as noted by Silva et al.^12^ in a survey conducted with 170 undergraduate students, of which 89.51% were in favor of the creation of this specialty. Araújo & Medeiros^13^ conducted a study with 143 students, of which 85.7% agreed with the creation of the specialty. This may be attributed to the small number of universities (only 6.9%) that included the subject in the curriculum of Dentistry courses, as evidenced in the present study.

Studies analyzing the curriculum of Dentistry courses in Brazil are lacking. The available literature highlights inadequacies such as the alienation of graduation courses from community problems^14^ and outdated curricula with subjects addressing specialties with few CFO-registered professionals, such as Temporomandibular Disorder,^15^ in addition to recently recognized qualifications, such as Hospital Dentistry.^16^

Soares et al.^9^ believe there should be more investment in the education of health professionals during initial training and continuing education. However, as noted in the present study, the process of including OD in the curriculum of both public and private universities has been heterogeneous, both in relation to the compulsory status of the subject and its workload. Universities that included the subject are mostly private, such as in the case of Minas Gerais, which accounts for 40% of all registered universities. However, these universities are all private, which implies greater compliance with legal determinations and recommendations from the Brazilian Ministry of Education regarding curriculum adequacy of pedagogical projects.^17^ Several subjects lack standardization regarding their workload, compulsory status, and curriculum, such as Implant Dentistry,^18^ Bioethics,^19^ Medical Emergencies,^20^ and Brazilian Sign Language.^21^ This issue may also be attributed to current requirements in the formal training of Dentistry professors, which should undergo a thorough review to evaluate the meaning of their role.^22^

Toassi et al.^23^ analyzed the process of updating the curriculum of a Dentistry course of a university in Southern Brazil and showed that this process requires continuous reconstruction in an organized way by a qualified team that can produce quality educational practices.^24^

Shulman^25^ found that professors should have the following skills: communication; planning ability; methodology conception and activity organization, including space organization; and selection and development of tasks.^25^ These factors represent a challenge in the formal training of health professionals, in which technical courses are still predominant.^26^

According to data from the CFO,^27^ only 1,100 professionals specialized in OD were registered in the CFO in 2010: 125 in the state of Minas Gerais, 64 in Espírito Santo, 395 in Rio de Janeiro, and 193 in São Paulo. This represents a low number of qualified professionals in the field (0.93%), which may be attributed to the shortage of qualified professors in OD, as well as the small number of Dentistry courses that include this subject in their curriculum.

A lack of qualified professionals teaching subjects related to the psychosocial field was reported by Pires & Shimizu,^28^ who found that 86.47% of professors teaching Bioethics-related subjects did not attend non-degree or postgraduate courses in Philosophy, Ethics, or Bioethics.^28^ A lack of standardization in the nomenclature of the subject was also observed. Because there is no standardization regarding the compulsory status of OD, as well as because of a lack of standardization, some universities teach OD as a topic of other subjects, as in the case of Bioethics and Forensic Dentistry.^29^

In view of the foregoing, missing data related to universities registered with the Brazilian Ministry of Education should be investigated to allow a more detailed analysis of the inclusion of OD in Dentistry courses, which could promote the elaboration of teaching guidelines for OD, as well as the creation of the Brazilian Society of Occupational Dentistry for greater recognition and appreciation of the importance of this specialty.

## CONCLUSIONS

The present study investigated the overall inclusion of OD in the curriculum of Dentistry courses in Southeast Brazil. Only a small percentage of universities (6.9%), mostly private, included the subject in the course curriculum, usually on a compulsory basis. Given the importance of this subject, more undergraduate Dentistry courses should include OD in their curriculum to train qualified professionals who are equipped to deal with work-related oral health issues.
